# Super-resolution CT Image Reconstruction Based on Dictionary Learning and Sparse Representation

**DOI:** 10.1038/s41598-018-27261-z

**Published:** 2018-06-11

**Authors:** Changhui Jiang, Qiyang Zhang, Rui Fan, Zhanli Hu

**Affiliations:** 1 Lauterbur Research Center for Biomedical Imaging, Shenzhen Institutes of Advanced Technology, Chinese Academy of Sciences, Shenzhen, 518055 China; 2 Shenzhen College of Advanced Technology, University of Chinese Academy of Sciences, Shenzhen, 518055 China

## Abstract

In this paper, a single-computed tomography (CT) image super-resolution (SR) reconstruction scheme is proposed. This SR reconstruction scheme is based on sparse representation theory and dictionary learning of low- and high-resolution image patch pairs to improve the poor quality of low-resolution CT images obtained in clinical practice using low-dose CT technology. The proposed strategy is based on the idea that image patches can be well represented by sparse coding of elements from an overcomplete dictionary. To obtain similarity of the sparse representations, two dictionaries of low- and high-resolution image patches are jointly trained. Then, sparse representation coefficients extracted from the low-resolution input patches are used to reconstruct the high-resolution output. Sparse representation is used such that the trained dictionary pair can reduce computational costs. Combined with several appropriate iteration operations, the reconstructed high-resolution image can attain better image quality. The effectiveness of the proposed method is demonstrated using both clinical CT data and simulation image data. Image quality evaluation indexes (root mean squared error (RMSE) and peak signal-to-noise ratio (PSNR)) indicate that the proposed method can effectively improve the resolution of a single CT image.

## Introduction

In recent years, low-dose computed tomography (CT) technologies, such as limited-angle reconstruction algorithms, have been widely studied by researchers to reduce radiation damage in patients^1^. However, both conventional analytic CT algorithms, such as filtered backprojection (FBP) and the simultaneous algebraic reconstruction technique (SART), have limitations in dealing with this problem^2^. FBP algorithms need a high sampling rate for the appropriate image quality, which results in a radiation dose increase. Although SART algorithms can improve the signal-to-noise ratio of the output images, these methods usually have a heavy computing burden and always lead to oversmoothed output images. These limitations of analytic CT algorithms and iterative algorithms usually degrade the image resolution of clinical CT data, and noisy low-resolution (LR) images may result in misdiagnosis^3,4^. Therefore, the utilization of super resolution (SR) based on dictionary learning and sparse representation is necessary to obtain high-resolution (HR) images and avoid excessive smoothing.

In the image processing domain, recovery has always been a subject of intense study^5,6^. SR reconstruction is a classical image recovery technique. SR methods can be broadly classified into the following three categories: interpolation-based methods, reconstruction-based methods, and example-based methods^6^. Interpolation-based SR has been proposed for various applications and typically reduces the required amount of computation^7,8^. However, such methods are not suitable for generating fine details and often introduce additional artifacts in practice. Reconstruction-based methods are always combined with one or more well-designed priors^9^ to estimate the details missed in the reconstruction process. These methods can obtain good results in preserving edges on the premise that a rational prior has been imposed^10^. Learning-based SR methods^11–13^ have been proposed in recent years, and these methods are considered the most promising algorithms in the SR domain. Learning-based SR approaches usually include a training stage and a testing stage, and through these two steps, a mapping relationship is established between LR-HR patch pairs of an external database and input LR patches^14,15^. Finally, the missing high-frequency details can be estimated.

Many learning-based SR methods have been proposed after years of development, and these methods have become the main approaches used in SR algorithms. Sparse representation-based methods are considered the most successful learning approaches. In sparse representation-based approaches, it is generally effective to obtain dictionaries by training over example signals using the dictionary learning process. Many researchers have focused on SR via sparse representation and trained dictionaries. M. Elad *et al*.^16^ suggested that sparse representation exhibits strong robustness to noise. Dong *et al*.^17^ used K-means clustering to organize training data into several clusters to compact dictionaries. Feng *et al*.^18^ employed K-space clustering to divide the signal space into subspaces and to extract the common elements to form a dictionary. Yu *et al*.^19^ used a composition of orthogonal basis functions based on specific signal structures to construct a structural dictionary. Yang *et al*.^20^ proposed a multiple geometric dictionary-based clustered sparse coding scheme, where the image patches are sparsely coded over different dictionaries.

Inspired by the success of image SR through sparse representation in these papers, we introduce a similar method to obtain HR CT images. This method uses appropriate sparse representations and trained dictionaries of LR CT images. We use the relationship between LR and HR image patches by assuming that these patches share the same sparse coefficients as their respective dictionaries, which are jointly obtained from a set of external training images. Additionally, in the process of SR imaging, we subject the output SR images to the proposed method for several iterations to enhance the image resolution.

It is generally known that due to an insufficient number of LR images and reconstruction constraints, the SR image reconstruction issue is an ill-posed problem. The aforementioned methods require enormous databases of HR and LR patch pairs and therefore require large amounts of calculations. In this paper, we use regularization methods to solve this problem. Therefore, this method relies on two coupled dictionaries, *D*_*l*_ and *D*_*h*_. *D*_*l*_ is trained on LR CT image data, and *D*_*h*_ is trained on HR image data. Both dictionaries are overcomplete^21^. To capture the co-occurrence prior, the sparse representation of the image patch pairs sampled from HR and LR images is determined. Finally, the sparse representation of an input LR CT image based on *D*_*l*_ is used to obtain an HR CT image from the *D*_*h*_ dictionary. Based on the linear relationships between the input and output data^22^, this method obviously improves the speed of the reconstruction algorithm.

To ensure that the image patch pairs have the same sparse representations, we train the two dictionaries simultaneously by concatenating them with proper normalization. The trained compact dictionaries are applied to both generic SR images. Compared to the regularization-based methods mentioned above, the proposed algorithm performs adaptive selection well, which leads to its superior ability to improve resolution and denoising. This work demonstrates that sparse prior information can be used to improve the quality of low-count CT reconstruction images and presents a novel approach in the CT field for solving the LR problem.

In conclusion, there are three main advantages of the proposed method. First, the proposed SR method is effective for not only LR images but also noisy images. Moreover, no additional training dictionaries are required for CT image denoising because the LR image patches subtract the mean pixel values used for the next dictionary training. This step creates a one-to-one correspondence between the LR patches and the relevant SR patches. Then, we jointly train two dictionaries for the LR and HR image patches and use the sparse representation coefficients of the LR image patch to generate the corresponding HR image patch. This operation guarantees that the HR dictionary represents textures of HR example patches and LR example patches that are noise free. Second, the proposed SR method can reduce the amount of calculations compared with that of traditional methods due to the utilization of a sparse-coding trained dictionary. Third, to obtain HR CT images, we perform several iteration steps until the output image is sufficiently close to an ideal image. We use the image quality root mean squared error (RMSE), peak signal-to-noise ratio (PSNR) and structural similarity index (SSIM) to evaluate image quality at this step.

This paper is organized as follows. Section II provides a detailed description of how the HR and LR image dictionaries are trained and describes the process of the image SR methods based on sparse representation. In Section III, we implement the algorithm with simulation phantom images and clinical images. Experimental results are compared with the output of existing methods. In Section IV, the major findings of this study are summarized.

## Methods and Materials

### SR based on sparse representation

We model the problem as follows, based on^14^. We generate an HR CT image *X* from a given LR CT image *Y* under the same conditions. To solve this problem, this work adopts two constraints.

One constraint is that HR image *X* should be consistent with LR image *Y* for the sparse reconstruction:

1 $$Y=SHX$$

where *H* is a sparse matrix, and *S* is the sampling matrix. In matrix *S*, we can determine the degradation of the geometric shift, blur, or downsample operator for image *X*.

The other constraint is that the small patches *x* segmented from HR CT image *X* can be sparsely represented in terms of the *D*_*h*_ dictionary as follows:

2 $$x\approx {D}_{h}\alpha \,{\rm{for}}\,{\rm{some}}\,\alpha \in {R}^{K}\,{\rm{with}}\,{\alpha }_{0}\ll K$$

where α is the sparse representation coefficient of patch *x* for input image *Y*. For this concept, sparse condition (3) is used to obtain the representation of each local patch. Then, using constraint Eq. (1) as the premise of this step, the representative results are used as the basis for recovering the entire image. Therefore, there are two models in the method: a local model and a global model. Using the local model, we train two dictionaries *D*_*l*_ and *D*_*h*_, which represent the textures of the images and cause the LR and HR CT images to have the same sparse representation. Then, according to *D*_*l*_, the process of finding the sparsest representation can be depicted as follows:

3 $$\min \,{\Vert \alpha \Vert }_{0}\,s.\,t.\,{\Vert F{D}_{l}{\boldsymbol{\alpha }}-Fy\Vert }_{2}^{2}\ll \varepsilon ,\,$$

where *F* is the feature extraction operator of the LR CT image. Because the details of an image often involve its high-frequency content, *F* is similar to a high-pass filter. The function of *F* is to ensure that the value of *α* calculated in Eq. (3) is closely related to the image being recovered, thus making the prediction more reasonable. The global model from constraint Eq. (1) is used to guarantee the robustness of the recovered image and can also suppress possible artifacts that arise from the local model.

Although Eq. (4) is NP-hard, it can be recovered by the ${\ell }^{1}$-norm when the coefficient ***α*** is sufficiently sparse^23–25^. The optimization is as follows:

4 $${\min }_{\alpha }{\Vert F{{\boldsymbol{D}}}_{l}{\boldsymbol{\alpha }}-F{\boldsymbol{y}}\Vert }_{2}^{2}+{\rm{\lambda }}{\Vert {\boldsymbol{\alpha }}\Vert }_{1}$$

where λ balances the sparsity of the solution and the fidelity of the observed image patch ***y***.

In Eq. (4), we use image patches in the calculation, making the compatibility between adjacent patches the most significant factor. Therefore, we modify Eq. (4) based on the one-pass algorithm, and the optimization is as follows:

5 $${\min }_{{\boldsymbol{\alpha }}}{\Vert \tilde{{\boldsymbol{D}}}{\boldsymbol{\alpha }}-\tilde{{\boldsymbol{y}}}\Vert }_{2}^{2}+\lambda {\Vert {\boldsymbol{\alpha }}\Vert }_{1}$$

Where $$\tilde{{\boldsymbol{D}}}=[\begin{array}{c}F{{\boldsymbol{D}}}_{l}\\ \beta P{{\boldsymbol{D}}}_{h}\end{array}]$$, $$\tilde{{\boldsymbol{y}}}=[\begin{array}{c}F{\boldsymbol{y}}\\ \beta {\boldsymbol{\omega }}\end{array}]$$, matrix P guarantees compatibility by extracting the overlap region, and ***ω*** contains the values of the adjacent reconstructed image upon overlap, *β* keeps the consistency between the HR images matching the input and neighborhood areas, and we also set *β* = 1. Then, when obtaining the solution α^*^, we can obtain the HR patches ***x***=**D**^2^α^*^.

Once we determine all of the calculated patches, we obtain ***X***_0_. To fulfill the sparse constraints of Eq. (2), we substitute ***X***_0_ into Eq. (1) and obtain

6 $${X}^{\ast }=\text{arg}{\min }_{{\boldsymbol{X}}}{\Vert SH{\boldsymbol{X}}-{\boldsymbol{Y}}\Vert }_{2}^{2}+c{\Vert {\boldsymbol{X}}-{{\boldsymbol{X}}}_{0}\Vert }_{2}^{2}$$

This solution can be solved using the gradient descent. The updated equation is

7 $${{\boldsymbol{X}}}_{t+1}={{\boldsymbol{X}}}_{t}+\nu [{H}^{T}{S}^{T}(Y-SH{{\boldsymbol{X}}}_{t})+c({\boldsymbol{X}}-{{\boldsymbol{X}}}_{0})]$$

where ***X***_*t*+1_ is the predicted HR image at the t + 1 iteration, and ***v*** is the step size of the gradient descent.

In these models, the primary problem is determining the sparse representation of the image. The most important step is to acquire a suitable overcomplete dictionary that fits Eq. (3). In this paper, we trained two related dictionaries, *D*_*l*_ and *D*_*h*_. These two dictionaries would make the related low- and high-resolution images have approximately the same sparse representation. These dictionaries are trained with the given CT image patch pairs ({$Y|Y={y}_{1},{y}_{2},\cdots ,{y}_{n}$} and {*X* |*X* = ${x}_{1}$,$\,{x}_{2}$, ...,$\,{x}_{m}$}), which can be formulated as follows:

8 $${\min }_{\{{{\boldsymbol{D}}}_{l},{{\boldsymbol{D}}}_{h},Z\}}{\Vert {{\boldsymbol{X}}}_{c}-{{\boldsymbol{D}}}_{c}\Vert }_{2}^{2}+\lambda (\frac{1}{M}+\frac{1}{N}){\Vert Z\Vert }_{1}$$

where $${{\boldsymbol{X}}}_{c}=[\begin{array}{c}\frac{1}{\sqrt{N}}{\boldsymbol{X}}\\ \frac{1}{\sqrt{M}}{\boldsymbol{Y}}\end{array}]$$, $${{\boldsymbol{D}}}_{c}=[\begin{array}{c}\frac{1}{\sqrt{N}}{{\boldsymbol{D}}}_{h}\\ \frac{1}{\sqrt{M}}{{\boldsymbol{D}}}_{l}\end{array}]$$, *M* and *N* are the dimension of *X* and *Y*, and ${\ell }^{1}$-norm ${\Vert Z\Vert }_{1}$ is included to enforce sparsity. The parameter *λ* is intended to balance the sparsity of the solution. By choosing an appropriate value of *λ*, we can balance the approximation error and the sparsity of ${\Vert Z\Vert }_{1}$.

### Dictionary training

**Figure 1:**
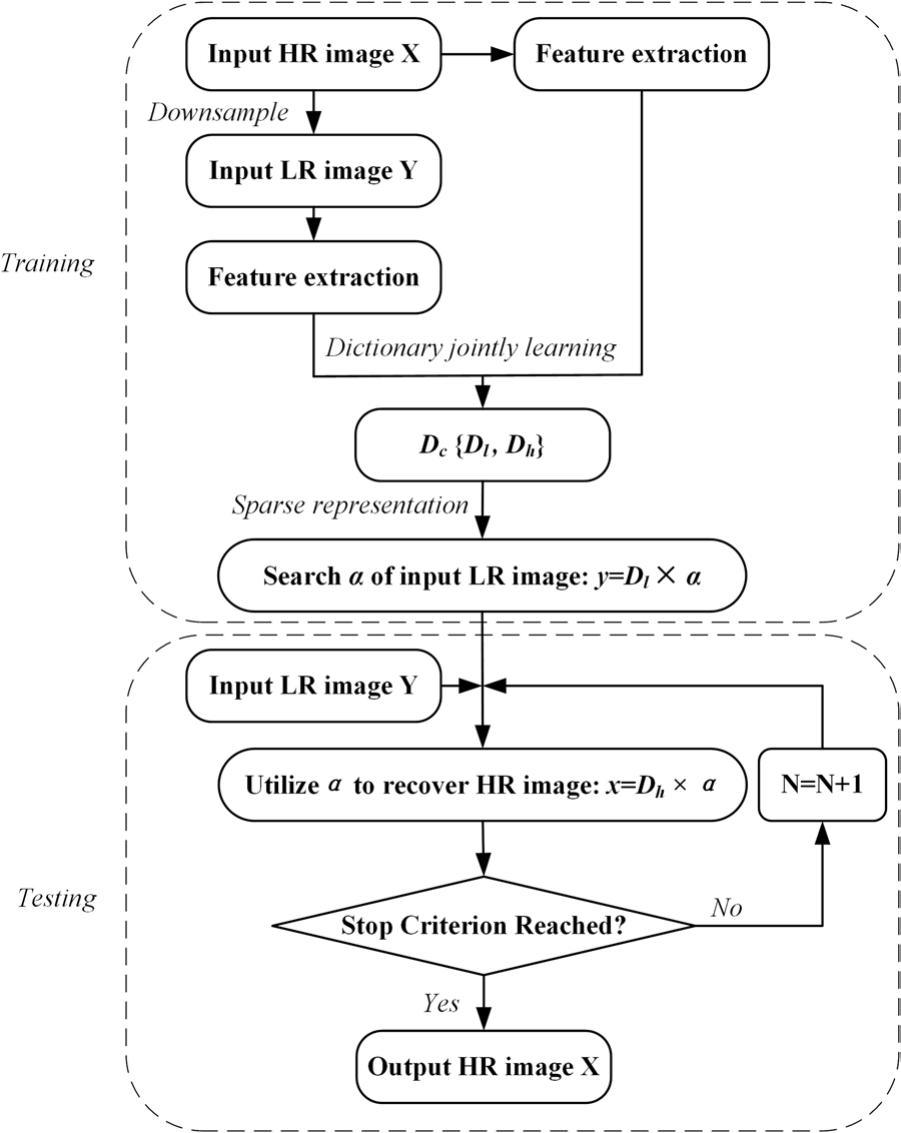
Flowchart of the proposed SR algorithm.

The proposed algorithm is composed of three stages. The first stage is the training stage; a set of dictionary LR and HR image pairs is trained. The second is the reconstruction stage, in which the best dictionary pair is selected to sparsely reconstruct the HR patches from their corresponding LR patches. The third is the iterative SR reconstruction stage, in which each reconstructed HR image is entered into the second stage to refine the image quality. A flowchart of the proposed SR algorithm is shown in Fig. 1.

The training stage requires a set of HR images and a set of LR images. The LR images are obtained by blurring with an average filter and downsampling the corresponding HR images. In addition, the LR images are magnified to have the same dimensions as the corresponding HR images using an interpolation operation. The interpolated LR images are used to extract features with a feature extraction filter. In this paper, we use the first- and second-order derivatives as the feature for the LR patch^26^ due to their simplicity and effectiveness. Then, these extracted features are used in the dictionary learning process and for sparse coding of the LR patches. Finally, LR and HR patches corresponding to the same spatial location are handled as pairs. To achieve sparse signal representations to better fit the data, the K-singular value decomposition (K-SVD) algorithm^27^ is used to train the dictionaries.

At the SR reconstruction stage, similar to the training stage, an LR image is first magnified to have the same dimensions as the corresponding HR image using bicubic interpolation. Features are extracted by applying feature extraction filters and are then reshaped into vector form. To ensure local consistency between the reconstructed patches, we apply a certain patch overlap with some pixels. Using the dictionary pair of the identified cluster, we first calculate the sparse representation coefficient vector of the corresponding HR feature vector over the cluster LR dictionary. Then, the HR patch is reconstructed by right-multiplying the cluster HR dictionary with the calculated sparse representation coefficient vector. Finally, an HR image is obtained by reshaping the reconstructed HR patches into two-dimensional form and then merging them.

### Ethics statements

All human experiments described in this work were approved by the Institutional Ethical Committee of Human Experimentation of Shenzhen Institutes of Advanced Technology (Chinese Academy of Sciences). The experiments were carried out strictly in accordance with relevant guidelines and regulations.

## Experimental Results

In this section, we demonstrate the performance of the proposed SR method. We chose 1000 high-quality images for each dictionary. The LR images used in the training or test stage were all generated from high-quality original HR images by directly blurring and downsampling. Noise was added to the blurred and downsampled test images. Before processing the LR image, we trained two dictionaries (*D*_*l*_ and *D*_*h*_) using 100,000 patch pairs randomly sampled from CT images collected from clinical hospitals and phantom simulations. To gain a balance between computation and image quality, we defined the size of the dictionary as 2048 atoms; this number of atoms can fully represent the features of the images without being too computationally intensive for our computer configuration. We empirically set the sparsity regularization *λ* = 0.4 and chose 5 × 5-pixel patches with an overlap of 4 pixels between adjacent patches. In our experiment, head data obtained by clinical CBCT and XCAT phantom simulation were used to evaluate the proposed method. We used RMSE, PSNR and SSIM to evaluate the output SR image quality.

### Digital phantom simulations

**Figure 2:**
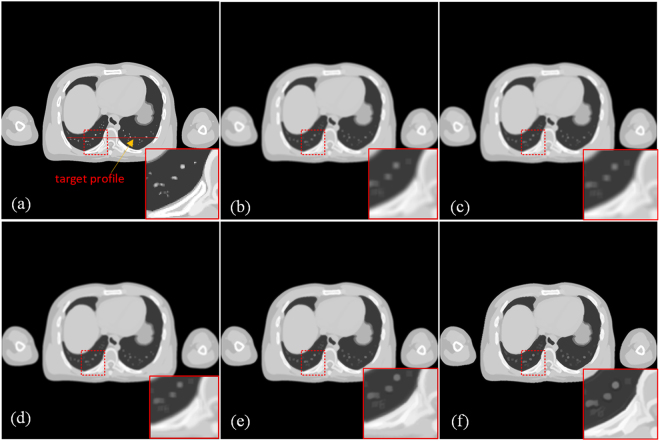
Comparison of SR reconstructed images of simulation CT images. (**a**) Original CT image. (**b**) Input LR image downsampled from (**a**). (**c**) Reconstructed image obtained using the bicubic interpolation method (PSNR = 29.9795, RMSE = 8.0829, SSIM = 0.9446). (**d**) Image reconstructed using the proposed method with 1 iteration (PSNR = 30.2243, RMSE = 7.8583, SSIM = 0.9468). (**e**) Image reconstructed using the proposed method with 5 iterations (PSNR = 31.8848, RMSE = 6.4908, SSIM = 0.9575). (**f**) Image reconstructed using the proposed method with 13 iterations (PSNR = 34.3108, RMSE = 4.9091, SSIM = 0.9723). All of the images are displayed in the same window. This display window is [0, 255].

**Figure 3:**
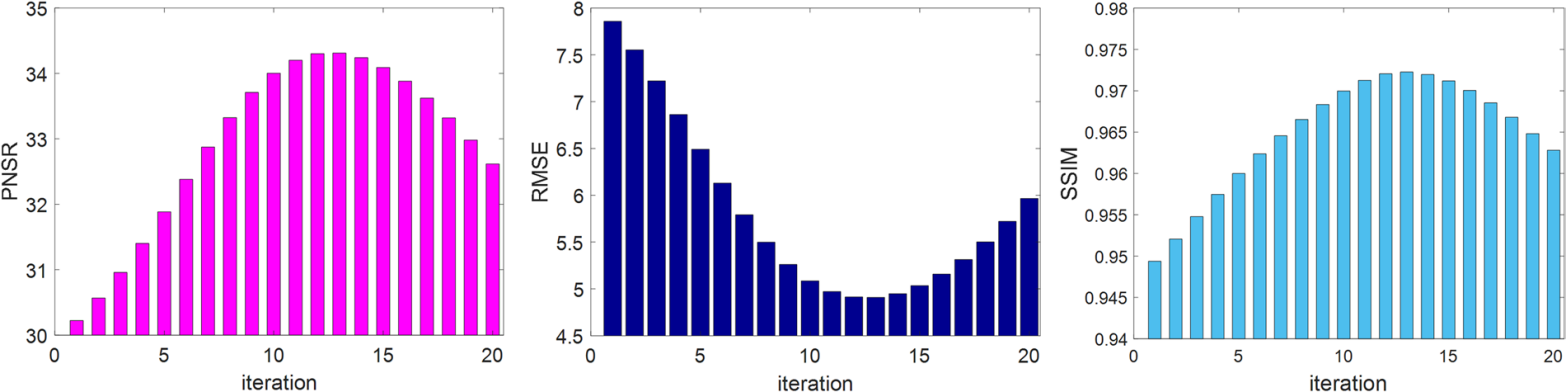
Changes in PSNR, RMSE and SSIM values with the number of iterations for the simulation experiment using the proposed method. After 13 iterations, the three indexes all reach an ideal value for the simulation images.

**Figure 4:**
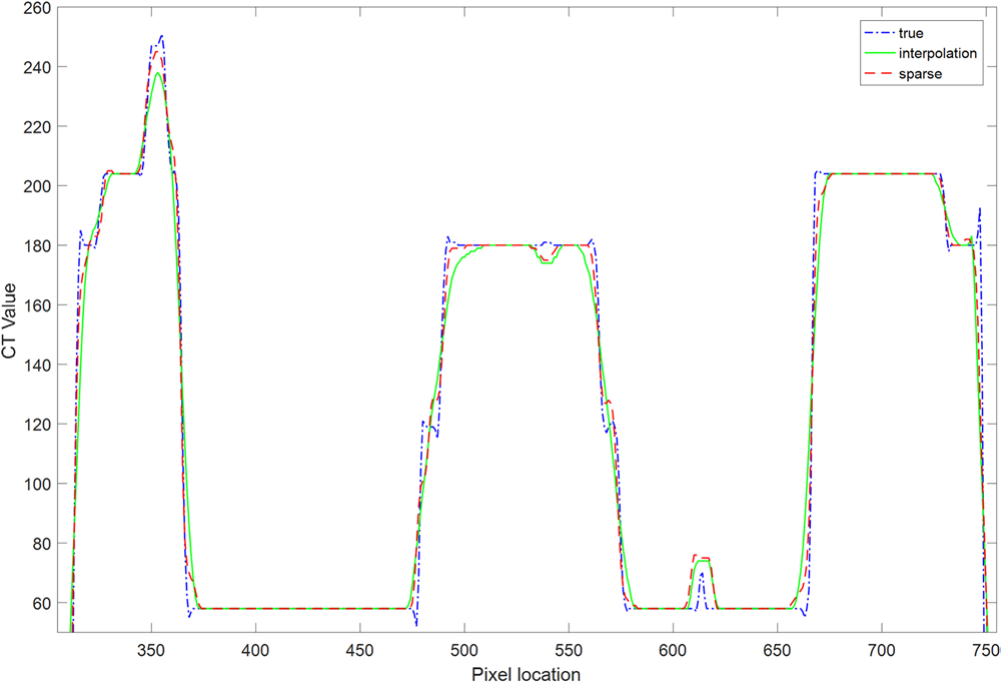
Profiles of different results shown in the 640^th^ row of Fig. 2. The true curve represents the profile of the original CT image in Fig. 2(a). The interpolation curve represents the profile of the reconstructed image obtained using the bicubic interpolation method in Fig. 2(c). The sparse curve represents the profile of the image reconstructed using the proposed method with 8 iterations from the original CT image, as shown in Fig. 2(f).

**Figure 5:**
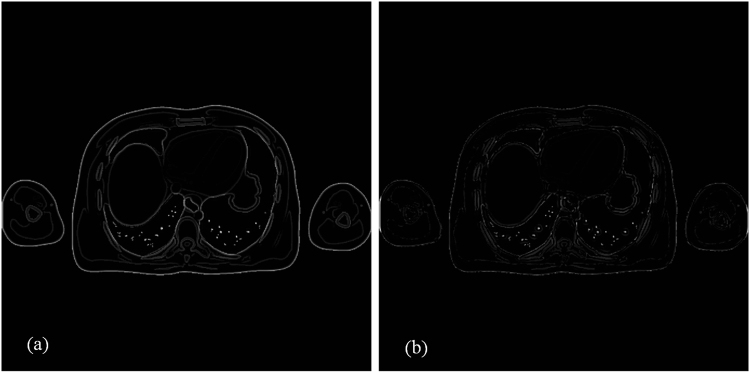
Residual images of the reconstructed results based on the bicubic interpolation method and the proposed method with 13 iterations for the simulation XCAT data. Both images are displayed in the same window.

In this simulation experiment, XCAT phantom data^28^ were used, as shown in Fig. 2. Similar to the processing of clinical images, one slice axial image of the phantom simulation was selected as the reference high-quality image, as shown in Fig. 2(a). The LR image in Fig. 2(b) was degraded from Fig. 2(a) for use as the input image. The reconstructed image in Fig. 2(c), achieved from the bicubic interpolation algorithm, was utilized and compared with the image in Fig. 2(f), which was processed using our method. The PSNR, RMSE and SSIM values of Fig. 2(c) were 29.9795, 8.0829 and 0.9446, respectively, while the corresponding values of Fig. 2(f) were 34.3108, 4.9091 and 0.9723. As shown in these images, compared with the image reconstructed using the bicubic interpolation method, the values of PSNR and RMSE improved by 14.44% and 39.27%, respectively. According to Fig. 3, it is obvious that the image quality index improved as the number of iterations increased, and the quality indexes PSNR, RMSE and SSIM reached their optimal values after 13 iterations. The profile images and residual images were also compared, as shown in Figs 4 and 5, respectively. We conclude that the proposed SR scheme has a clear performance advantage over the standard bicubic scheme.

### Clinical CBCT data experiments

To evaluate the algorithms in a more realistic situation, raw sinogram data were acquired using a commercial dental CT scanner (produced by Zhongke Tianyue Technology Co. Ltd., model ZCB-100). We used two strategies to test the performance and robustness of the proposed SR method. In the first strategy, we used the degraded clinical data as the input LR image. In the second strategy, the downsampled reconstruction data were used as the corresponding input image.

### Degraded clinical CBCT data restoration

**Figure 6:**
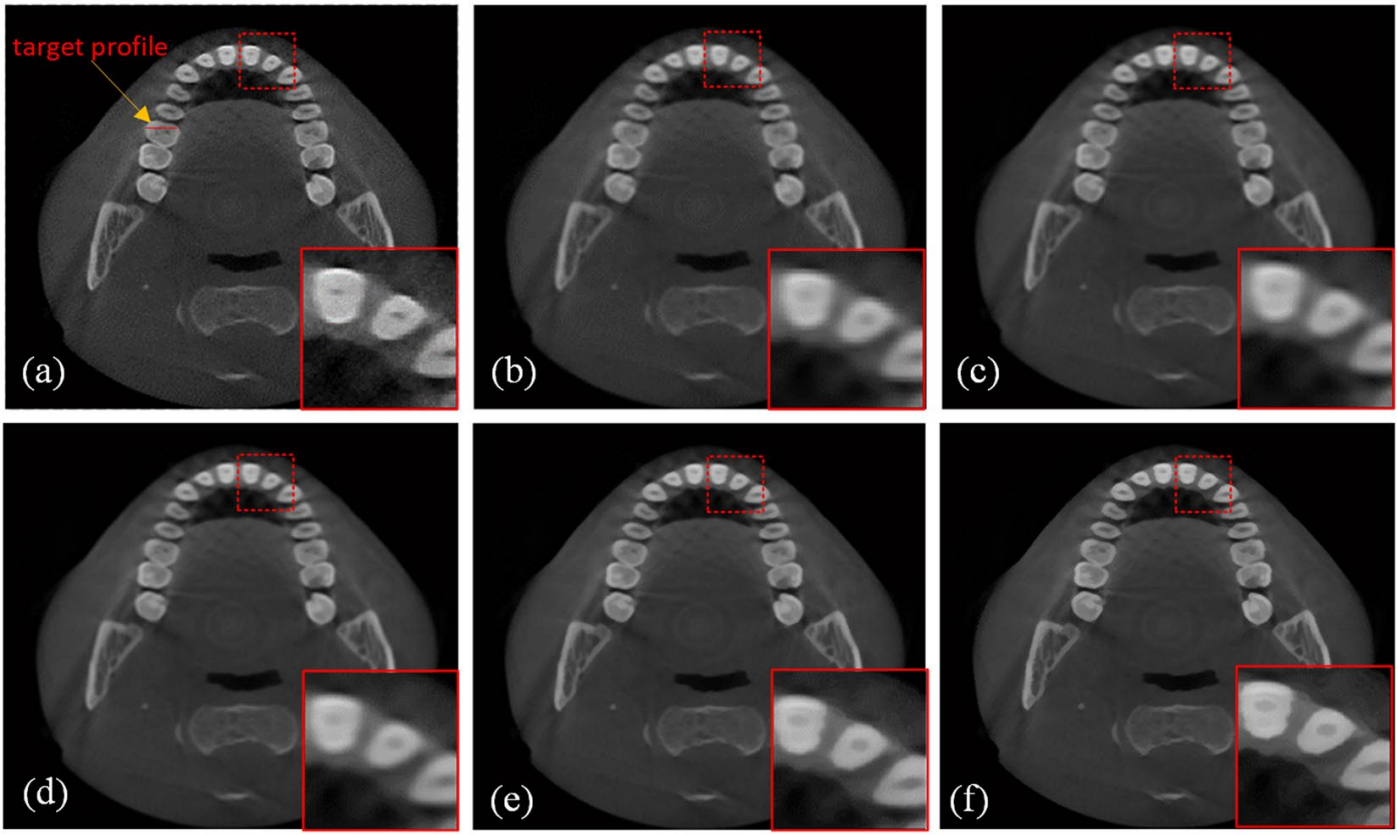
Comparison of SR reconstructed images of dental CT images. (**a**) Original CT image. (**b**) Input LR CT image degraded from (**a**). (**c**) Reconstructed image obtained using the bicubic interpolation method (PSNR = 35.0198, RMSE = 4.5243, SSIM = 0.8793). (**d**) Image reconstructed using the proposed method with 1 iteration (PSNR = 35.181, RMSE = 4.4411, SSIM = 0.8768). (**e**) Image reconstructed using the proposed method with 5 iterations (PSNR = 35.9691, RMSE = 4.0559, SSIM = 0.8798). (**f**) Image reconstructed using the proposed method with 8 iterations (PSNR = 36.1571, RMSE = 3.9691, SSIM = 0.8806). All of the images are displayed in the same window. This display window is [0, 255].

**Figure 7:**
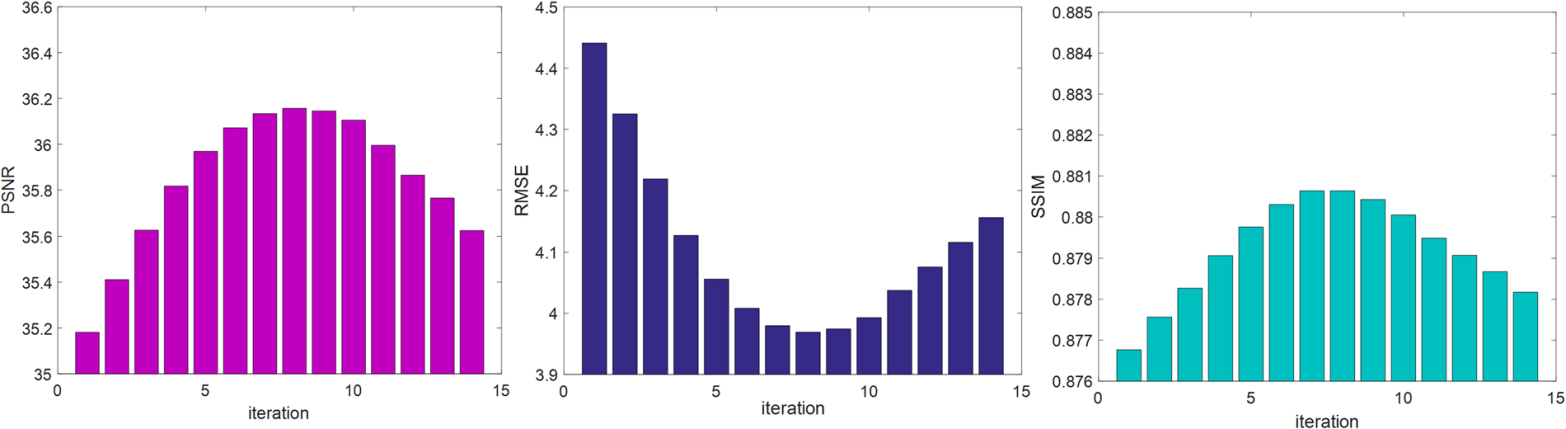
Changes in PSNR, RMSE and SSIM values with the number of iterations using the proposed method. After 8 iterations, the three indexes all reach ideal values for the dental SR images.

The input image was degraded by average filter in this section. After this operation, we obtained the blurry LR input image, and we then restored the input image using the proposed SR method and the bicubic interpolation method. Figure 6 compares the image quality of these two methods. The small images in the lower right corner are enlarged regions indicated by the red square. The performance was evaluated using PSNR, RMSE and SSIM. Figure 6(a) shows the original high-quality CT image for reference, and Fig. 6(b) shows an input LR CT image degraded from (a). Figure 6(c) presents an image reconstructed using the bicubic interpolation method (PSNR = 35.0198, RMSE = 4.5243, SSIM = 0.8793). Figure 6(d) provides an image reconstructed using the proposed method with 1 iteration (PSNR = 35.181, RMSE = 4.4411, SSIM = 0.8768). Figure 6(e) presents an image reconstructed using the proposed method with 5 iterations (PSNR = 35.9691, RMSE = 4.0559, SSIM = 0.8798), and Fig. 6(f) shows an image reconstructed using the proposed method with 8 iterations (PSNR = 36.1571, RMSE = 3.9691, SSIM = 0.8806). The improvement in the two image quality indexes is clear: 3.25% improvement for PSNR and 12.27% for RMSE. Because the proposed method follows an iterative process, we can see that the image quality index improved as the iteration number increased, and the quality indexes PSNR, RMSE and SSIM reached ideal values after 8 iterations, as shown in Fig. 7.

**Figure 8:**
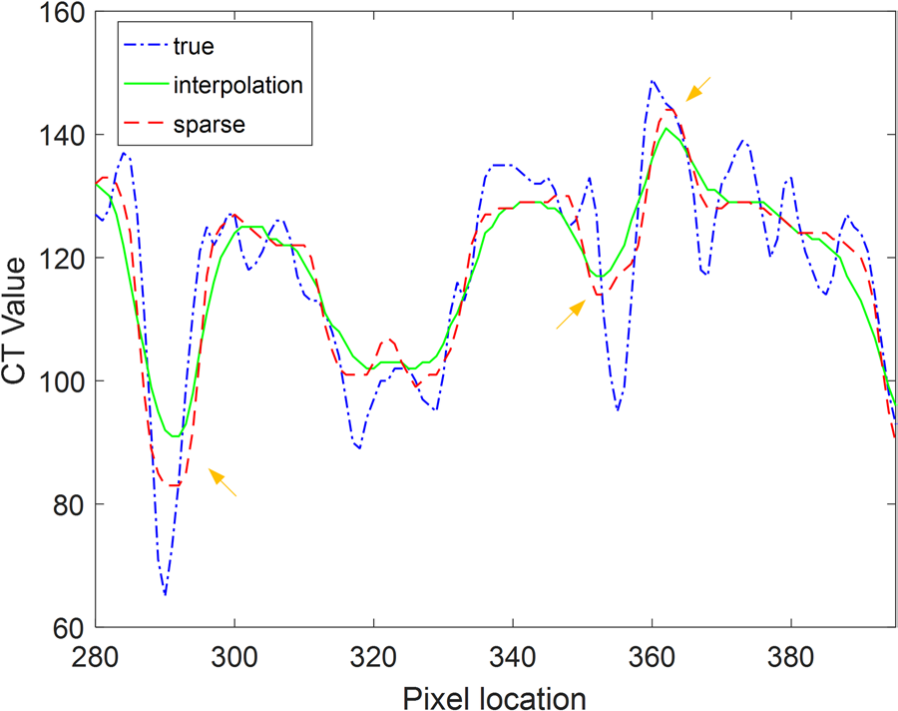
Profiles of different results for the horizontal line labeled in Fig. 6(a) in the 300^th^ row of the clinical CT images. The true curve represents the profile of the original CT image. The interpolation results represent the profile of the reconstructed image achieved using the bicubic interpolation method. The sparse curve represents the profile of the image reconstructed using the proposed method with 8 iterations.

**Figure 9:**
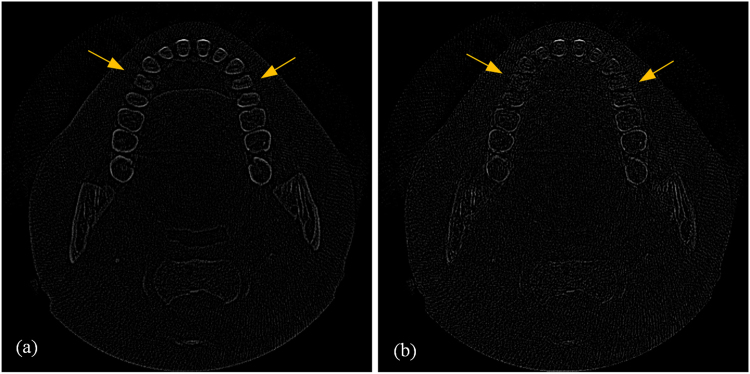
Residual images of the reconstructed results based on the bicubic interpolation method and the proposed method with 8 iterations. Both images are displayed in the same window.

To quantitatively evaluate the reliability of the proposed SR method, the profile images and the residual images of the reference image were also compared, as shown in Figs 8 and 9, respectively. The results show that the proposed method can help achieve image quality superior to that of the standard method.

**Figure 10:**
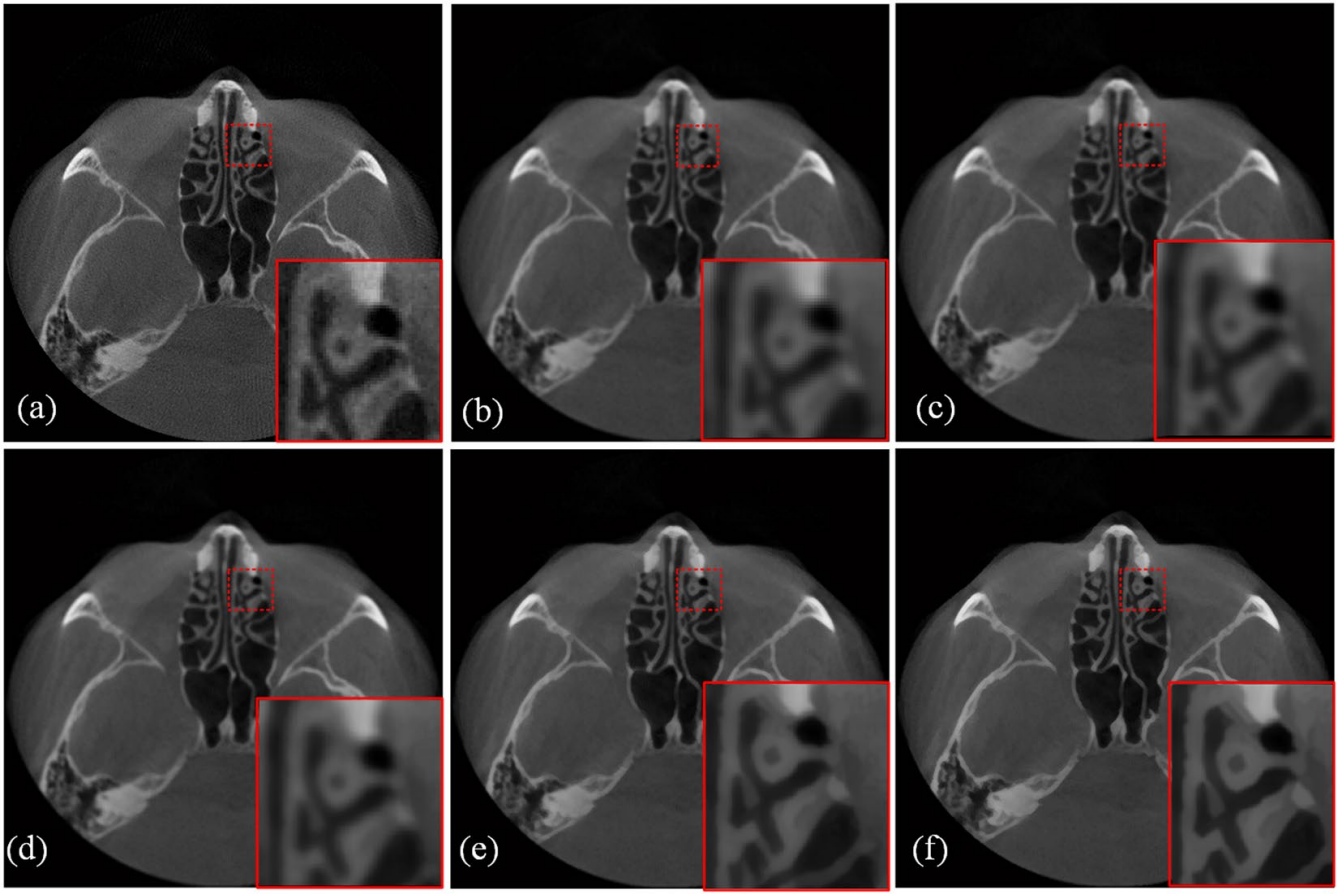
Comparison of SR reconstructed images for another slice of the brain CT images. (**a**) Original CT image. (**b**) Input LR CT image downsampled from (**a**). (**c**) Reconstructed image obtained using the bicubic interpolation method (PSNR = 33.1202, RMSE = 5.6302). (**d**) Image reconstructed using the proposed method with 1 iteration (PSNR = 33.2434, RMSE = 5.5509). (**e**) Image reconstructed using the proposed method with 5 iterations (PSNR = 33.8063, RMSE = 5.2027). (**f**) Image reconstructed using the proposed method with 7 iterations (PSNR = 33.8809, RMSE = 5.1581). All images are displayed in the same window. This display window is [0, 255].

Additionally, we applied the proposed approach to another slice image of the clinical head data, as shown in Fig. 10. The image quality of the output image was significantly improved.

### Downsampled clinical CBCT data restoration

First, the CT projections were gathered in the full-angle and normal-dose scan model to reconstruct the reference high-quality image. In particular, instead of scanning the patient twice, we simulated the corresponding low-dose sinogram data by choosing data with certain numbers of projections at equal angle intervals in a limited angle from the normal-dose sonogram data. In this section, the input image was reconstructed using the ART-TV method with 120 projections sampled in 360° at uniform angle intervals. We chose one slice of the reconstructed low-dose images of the dental CT as the input low-quality image for the proposed SR method and the bicubic interpolation method. Finally, we compared images restored using these two methods. We also used RMSE, PSNR and SSIM to evaluate the image quality in this step.

**Figure 11:**
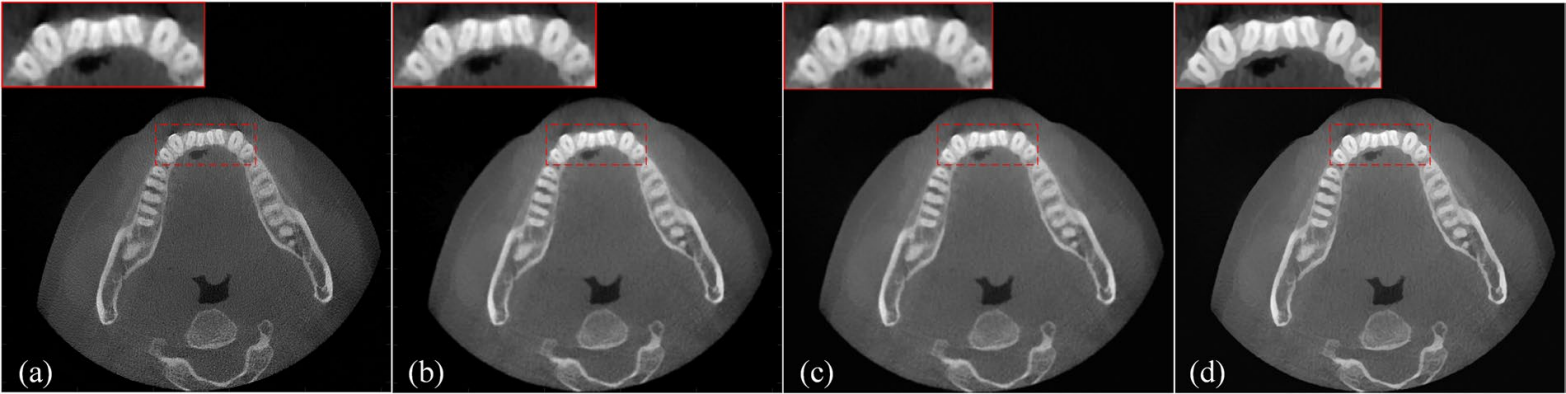
Comparison of restored images for one downsampled slice of the dental CT images. (**a**) Reference full-sampling CT image reconstructed using the ART-TV method with 360 projections. (**b**) Input LR CT image reconstructed using the ART-TV method with 120 projections. (**c**) Restored image using the bicubic interpolation method. (**d**) Image reconstructed using the proposed method with 4 iterations. All images are displayed in the same window. These images are all in 16-bit format, and the display window is [0, 10000].

Figure 11(a) shows the reference high-quality CT image, and Fig. 11(b) shows an input LR CT image reconstructed by 120 projections. Figure 11(c) presents an image reconstructed using the bicubic interpolation method. Figure 6(d) provides an image reconstructed using the proposed method with 4 iterations. The small images in the upper left corner are enlarged regions indicated by the red square for the corresponding images. It is obvious that the performance of our proposed method is better than that of the bicubic interpolation method in the visual sense.

In Table 1, we list the RMSE, PSNR and SSIM values of the restored images. As shown in the table, the proposed SR method using dictionary learning and sparse representation yielded smaller RMSE values and larger PSNR and SSIM values.

**Table 1:** Quantified comparisons of image results restored using the bicubic interpolation method and the proposed method for low-dose image restoration.

Index	Input	Interpolation	Proposed
RMSE	327.0048	268.356	256.76
PSNR	31.8304	33.5473	33.931
SSIM	0.9759	0.9843	0.9854

In summary, in this section, the experimental results of the XCAT phantom simulation data and clinical CBCT data demonstrate that the proposed SR method based on dictionary learning and sparse representation yields images with better qualitative and quantitative performance.

## Conclusions

In this paper, a single-CT image SR reconstruction scheme is proposed. This SR reconstruction scheme is based on sparse representation theory and dictionary training of a set of LR and HR image patch pairs. The quality of the SR reconstruction images greatly depends on whether the employed sparse domain can adequately represent the target image by jointly training the LR and HR dictionaries. We establish a sparse representation for each patch of LR images and ensure that similarity exists between the LR and HR local patches. The coefficients in the LR domain are utilized to reconstruct the HR counterpart. In the process of HR image reconstruction, we introduce several iteration operations to improve image quality. We find that the quality of the reconstructed HR image is improved after several operational iterations. The results of the designed experiments demonstrate that the proposed algorithm can improve the resolution of noisy images and yield smaller RMSE and larger PSNR and SSIM values.
